# Robust metal ion-chelated polymer interfacial layer for ultraflexible non-fullerene organic solar cells

**DOI:** 10.1038/s41467-020-18373-0

**Published:** 2020-09-09

**Authors:** Fei Qin, Wen Wang, Lulu Sun, Xueshi Jiang, Lin Hu, Sixing Xiong, Tiefeng Liu, Xinyun Dong, Jing Li, Youyu Jiang, Jianhui Hou, Kenjiro Fukuda, Takao Someya, Yinhua Zhou

**Affiliations:** 1 Wuhan National Laboratory for Optoelectronics, Huazhong University of Science and Technology, Wuhan, 430074 China; 2 Beijing National Laboratory for Molecular Sciences, State Key Laboratory of Polymer Physics and Chemistry, Institute of Chemistry, Chinese Academy of Sciences, 100190 Beijing, China; 3 Thin-Film Device Laboratory & Center for Emergent Matter Science (CEMS), RIKEN, 2-1 Hirosawa, Wako, Saitama 351-0198, Japan; 4 Department of Electrical Engineering and Information Systems, The University of Tokyo, 7-3-1 Hongo, Bunkyo-ku, Tokyo 113-8656, Japan

**Keywords:** Solar energy, Materials for devices

## Abstract

Achieving high power conversion efficiency and good mechanical robustness is still challenging for the ultraflexible organic solar cells. Interlayers simultaneously having good mechanical robustness and good chemical compatibility with the active layer are highly desirable. In this work, we present an interlayer of Zn^2+^-chelated polyethylenimine (denoted as PEI-Zn), which can endure a maximum bending strain over twice as high as that of ZnO and is chemically compatible with the recently emerging efficient nonfullerene active layers. On 1.3 μm polyethylene naphthalate substrates, ultraflexible nonfullerene solar cells with the PEI-Zn interlayer display a power conversion efficiency of 12.3% on PEDOT:PSS electrodes and 15.0% on AgNWs electrodes. Furthermore, the ultraflexible cells show nearly unchanged power conversion efficiency during 100 continuous compression-flat deformation cycles with a compression ratio of 45%. At the end, the ultraflexible cell is demonstrated to be attached onto the finger joint and displays reversible current output during the finger bending-spreading.

## Abstract (web summary)

Simultaneously achieving high efficiency and mechanical robustness is challenging for ultraflexible organic solar cells. Here, Qin et al. present a robust interlayer of Zinc-chelated polyethylenimine (PEI-Zn) to facilitate the demonstration of efficient and mechanically robust ultraflexible solar cells.

## Introduction

Organic solar cells (OSC) have been attracting great attentions due to their easy processing and increasing power conversion efficiency (PCE)^1–11^. Comparing to inorganic counterparts, the organic and polymer semiconductors have excellent mechanical flexibility which enables the fabrication of ultraflexible and stretchable organic optoelectronic devices^12–14^. The ultraflexible OSCs are attractive for the integrated applications in intelligent robots, electronic skin, and Internet of Things^15–22^. In 2011, the Bao group reported the stretchable OSCs on pre-stretched polydimethylsiloxane (PDMS) with poly(3-hexylthiophene):phenyl-C61-butyric acid methyl ester (P3HT:PCBM) active layer, obtaining a PCE of 2.0%^23^. Later, ultraflexible OSCs are reported on ultrathin polymer substrates (such as polyethylene terephthalate (PET), parylene, etc). The ultrathin OSCs can be transferred onto the pre-stretched elastomer to realize the stretchability. For example, Kaltenbrunner et al. reported an ultrathin OSC fabricated on 1.4-μm-thick PET and achieved a PCE of 4.2%^24^. Recently, the Someya group fabricated the ultrathin solar cells sandwiched between two parylene substrates^25,26^. Their PCE reaches about 10% and the cells show good thermal stability and water-proof properties.

Although the attractive features of the ultraflexible solar cells have been demonstrated, mechanical durability of the cells during the continuous deformation cycling at a micrometer-scale bending radius is still challenging. To obtain good mechanical robustness, all the functional layers (including electrodes, interlayers, and the active layer) are required to have good mechanical flexibility. The active layer is based on polymer-based mixture that has good mechanical flexibility. Poly(3,4-ethylenedioxythiophene): polystyrene sulfonate (PEDOT:PSS) and silver nanowires (AgNWs) electrodes are good options as flexible electrodes. Thus, proper design of the interlayers and electrodes is important. Typically used ZnO interlayer tends to crack under high bending strain that is detrimental to the mechanical robustness of the ultraflexible OSCs^27^. A mechanically robust interlayer is needed to achieve good deformation cycling durability. Amorphous polymer-based interlayer could meet the request due to their good mechanical flexibility^27,28^. Amine-containing polymer polyethylenimine (PEI) has been used to produce electron-collecting interface in fullerene solar cells^29^. But, high-performance nonfullerene acceptors tend to react with PEI. The reaction destroys their chemical and electronic structure of the acceptor^30,31^ and results in poor performance. Previously, we proposed a protonation strategy to suppress the reaction by changing the processing solvent from alcohol to water for PEIE^31^. This strategy only works well with the indium tin oxide (ITO) electrode which has poor mechanical robustness, but not with the flexible PEDOT:PSS electrode or AgNWs electrode. Protonated PEIE is an insulator and its thickness should be thin (about 10 nm) to ensure efficient electron extraction. It cannot be used to smoothen the rough surface of AgNWs. Therefore, it is important to develop interlayers that can endure high mechanical strain and be simultaneously compatible with the nonfullerene active layers and flexible electrode to fabricate ultraflexible OSCs with high PCE and good mechanical robustness.

In this work, we present a robust low–work function interlayer of Zinc ion (Zn^2+^) chelated PEI (denoted as PEI-Zn) for efficient ultraflexible nonfullerene OSCs. The PEI-Zn can endure a maximum bending strain over twice as high as that of ZnO. Simultaneously, the chelation of Zn^2+^ with the PEI reduces the chemical reactivity of PEI, and therefore the reaction between the PEI and the nonfullerene active layer is suppressed. The PEI-Zn can work efficiently as the interlayer with the flexible electrodes of PEDOT:PSS and AgNWs to realize ultraflexible OSCs. With the PEI-Zn, ultraflexible OSCs show a PCE of 12.3% with PEDOT:PSS electrode and a PCE of 15.0% with AgNWs. The cells show good mechanical durability during the continuous deformation cycling with a compression ratio of 45%.

## Results

### Structure and photovoltaic performance of ultraflexible OSCs

**Fig. 1:**
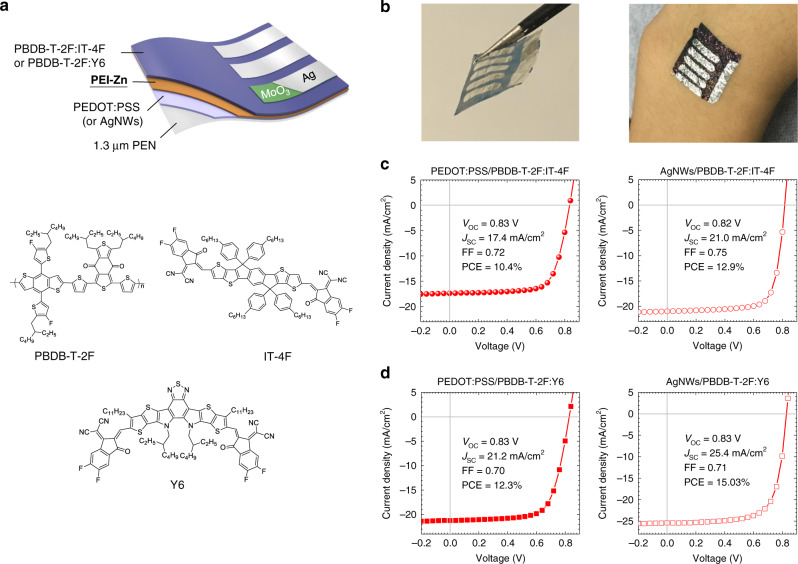
Device structure and photovoltaic performance of the ultraflexible OSC. **a** Device structure of the ultrathin solar cells and chemical structure of the nonfullerene active layer; A Zn^2+^-chelated polyethylenimine (PEI-Zn) is developed as the electron-transporting layer (ETL). **b** Pictures of the fabricated ultrathin OSCs; the picture on the right is a cell attached on the wrist. **c**, **d** *J*-*V* characteristics of the ultrathin devices with conducting polymer (PEDOT:PSS) or Ag nanowires as the transparent electrodes. Two different nonfullerene active layers of PBDB-T-2F:IT-4F and PBDB-T-2F:Y6 were used in the ultraflexible OSCs.

Figure 1 shows the device structure of the inverted ultrathin OSCs (PEN/PEDOT:PSS or AgNWs/PEI-Zn/active layer/MoO_3_/Ag). 1.3-μm-thick polyethylene naphthalate (PEN) is used as the substrate. PEDOT:PSS and AgNWs is used as the bottom transparent electrode. Two nonfullerene active layers of PBDB-T-2F:IT-4F^32,33^ and PBDB-T-2F:Y6^34^ are tried in the ultraflexible OSCs. Their chemical structures are shown in Fig. 1a. PEI-Zn interlayer is deposited between the bottom flexible electrode and the nonfullerene active layer for electron collection. The PEI-Zn is the key part of this work. Design and properties of PEI-Zn will be included in the next section. Pictures of the fabricated ultrathin nonfullerene solar cells are shown in Fig. 1b.

Figures 1c, d shows the current density-voltage (*J-V*) characteristics of the four different types of ultrathin OSCs: with active layer PBDB-T-2F:IT-4F or PBDB-T-2F:Y6 on PEDOT:PSS or AgNWs electrodes, respectively. When PBDB-T-2F:IT-4F is used as the active layer (Fig. 1c), cells with PEDOT:PSS electrode show a PCE of 10.4% (*V*_OC_ = 0.83 V, *J*_SC_ = 17.4 mA cm^−2^, FF = 0.72) and cells with AgNWs electrode show a PCE of 12.9% (*V*_OC_ = 0.82 V, *J*_SC_ = 21.0 mA cm^−2^, FF = 0.75). When PBDB-T-2F:Y6 is used as the active layer (Fig. 1d), cells with PEDOT:PSS electrode show a PCE of 12.3% (*V*_OC_ = 0.83 V, *J*_SC_ = 21.2 mA cm^−2^, FF = 0.70) and cells with AgNWs electrode show a PCE of 15.0% (*V*_OC_ = 0.83 V, *J*_SC_ = 25.4 mA cm^−2^, FF = 0.71). The cells on the AgNWs electrode (sheet resistance of 30 ohm sq^−1^) show higher *J*_SC_ than those on PEDOT:PSS electrode (sheet resistance of 110 ohm sq^−1^) due to the lower transmittance of the PEDOT:PSS film (Supplementary Fig. 1, the transmittance spectra measured with an integrating sphere). This is consistent with the external quantum efficiency (EQE) results where lower EQE values were observed in the long-wavelength region for cells with PEDOT:PSS electrodes (Supplementary Fig. 2). The output current remains 98% of the initial current after 6 h continuous illumination (Supplementary Fig. 3). PCE distribution histograms of individual cells with different electrodes and active layers are displayed in Supplementary Fig. 4. The device stability in air (with 100-nm Al_2_O_3_ encapsulation by atomic layer deposition, relative humility: 15%) is also tested. The devices remain over 80% of its initial efficiency under 200-h continuous illumination (Supplementary Fig. 5). There is no hysteresis observed in the *J*-*V* characteristics of cells when scanned in forward and reverse directions (Supplementary Fig. 6). 1-cm^2^ ultrathin solar cells are also fabricated on PEN substrates with AgNWs electrodes (with PBDB-T-2F:IT-4F active layer). The cell shows *V*_OC_ = 0.85 V, *J*_SC_ = 19.6 mA cm^−2^, FF = 0.67 and PCE = 11.2% which is comparable to the 1-cm^2^ reference cell on glass substrate/ITO (Supplementary Fig. 7). The performance of the 1-cm^2^ cells validates the effectiveness and reproducibility of the PEI-Zn interlayer for efficient electron collection.

### Chemical compatibility and mechanical property of PEI-Zn

**Fig. 2:**
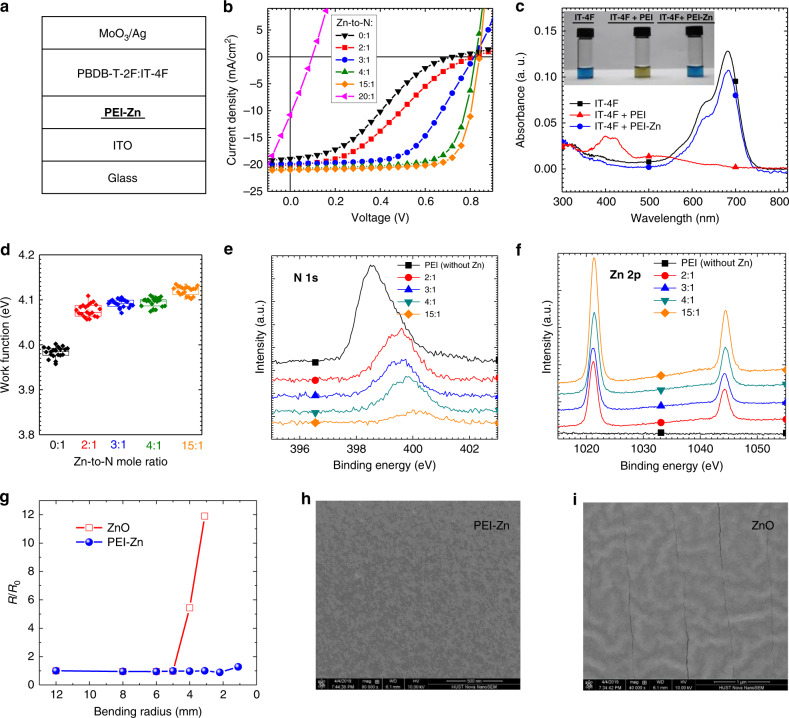
Properties and evaluation of PEI-Zn as an electron-transporting layer in OSCs. **a** Structure of OSC devices used to evaluate the performance of PEI-Zn as an electron-transporting layer; **b** *J*-*V* characteristics of the devices with PEI-Zn ETL containing different Zn-to-N mole ratios; Zn-to-N mole ratio is shown in the legend. **c** Absorption spectra of three solutions: IT-4F, IT-4F mixed with PEI, and IT-4F mixed with PEI-Zn solution. The inset shows the pictures of the three solutions. 100 μL 0.1 wt.% PEI and 100 μL PEI-Zn (Zn-to-N 15:1) were added to 1 mL 0.02 mg mL^−1^ IT-4F solution to study the reaction between them, respectively. **d** Work function of the PEI-Zn containing different Zn-to-N mole ratios; Boxes, square symbols, and horizontal bars indicate 25/75 percentile, mean, and min/max values, respectively. X-ray photoelectron spectroscopy of the PEI-Zn films containing different Zn-to-N ratios: **e** N 1 s; **f** Zn 2p. **g** Strain test on the PEI-Zn and ZnO films deposited on 175-µm thick PES substrates. Resistance ratio of the films after bending (*R*) over the initial value before bending (*R*_0_) as a function of the bending radius. SEM images the films on PES substrates after 500 times continuous bending with a bending radius of 4 mm: **h** PEI-Zn film; **i** ZnO film.

Development of PEI-Zn is the key part of the high-performance ultraflexible nonfullerene OSCs. To study the chemical compatibility of the PEI-Zn and the nonfullerene active layer, devices with a structure glass/ITO/PEI-Zn/PBDB-T-2F:IT-4F/MoO_3_/Ag (Fig. 2a) were fabricated. Different content of Zn^2+^ was added to optimize the PEI-Zn interlayer. First, PEI (without Zn^2+^) is used as the interfacial layer in the nonfullerene OSCs. The cell shows poor performance with a pronounced “S” shape in the *J-V* characteristics (Fig. 2b). Based on our previous study of the reaction between PEIE and IEICO-4F nonfullerene acceptor, it can be inferred that the PEI can chemically react with the IT-4F^31^. When PEI is added into the IT-4F chlorobenzene solution, the color of the IT-4F changed from dark blue to brown (Fig. 2c). Absorption band between 500 and 750 nm disappears (Fig. 2c). Combining ^1^H Nuclear magnetic resonance (NMR) study (Supplementary Fig. 8) and the mass spectroscopy (Supplementary Fig. 9), it is suggested that the amine reacts with the C=C linkage moiety through the Michael addition reaction that destroy the chemical and electronic structure of IT-4F^35^. The structure of PEI is complicated, and thus 1H NMR signals of the mixed solution of PEI and IT-4F are difficult to recognize. The absorption change of IT-4F solutions is similar when adding PEI and ethanolamine. So we used ethanolamine as the model compound to study the reaction. That leads to poor device performance. PEI was also added into the Y6 solution to study the reaction between Y6 and PEI. As shown in Supplementary Fig. 10a, color of Y6 solution changes from blue to light blue after adding PEI solution. Absorption band of Y6 between 600 and 800 nm becomes weaker. Meanwhile, solar cell with a structure of ITO/PEI/PBDB-T-2F:Y6/MoO_3_/Ag displays a low FF of 0.45 and a poor PCE of 6.08% (Supplementary Fig. 10b).

To suppress the reaction between PEI and the nonfullerene acceptor IT-4F, Zn^2+^ is introduced to chelate with the PEI by adding zinc acetate into the PEI solution. Zn^2+^ is selected to chelate with the PEI because Zn^2+^ has strong chelation properties with nitrogen atoms (such as in the compounds of Zinc porphyrin^36^, Zinc phthalocyanine^37^) and hydroxyl functional groups^38^. After the PEI is chelated with the Zn^2+^, the chemical reactivity of the PEI is reduced. As shown in Fig. 2c, the IT-4F solution mixed with the Zn^2+^ chelated PEI shows similar color and absorption spectra to the pristine IT-4F solution. The similar phenomenon is observed when PEI-Zn is added into the Y6 solution (Supplementary Fig. 10a). Note that the Zn chelated PEI is different from the mixture of PEI and ZnO precursor which will react with IT-4F solution. The color of the IT-4F solution changes when the PEI:ZnO precursor is mixed with IT-4F solution (Supplementary Fig. 11). For the solar cell performance, the ‘S’ shape in the *J-V* characteristics gradually disappears as the content of Zn^2+^ increases (Fig. 2b). The photovoltaic parameters are summarized in Supplementary Table 1. The device with pure PEI displays a pronounced ‘S’ shape with a low FF of 0.30. When the Zn-to-N mole ratio comes to 4:1, the “S” shape in *J*-*V* curve disappears with a high performance (*V*_OC_ = 0.82 V, *J*_SC_ = 20.7 mA cm^−2^, FF = 0.73 and PCE = 12.4%). The device performance is further improved with the mole ratio of Zn^2+^:N of 15:1 (*V*_OC_ = 0.84 V, *J*_SC_ = 20.8 mA cm^−2^, FF = 0.76 and PCE = 13.3%). As the content of Zn(CH_3_COO)_2_ continues to increase, the mixed solution gets cloudy because the Zn(CH_3_COO)_2_ cannot be fully coordinated with the PEI and dissolved. In addition to the zinc acetate, zinc acetylacetonate is also used to provide zinc source to coordinate with the PEI. Devices based on PEI-Zn with different ratios of zinc acetylacetonate are also evaluated. The performance shows a similar trend to the case of zinc acetate used as the zinc source (Supplementary Fig. 12). The photovoltaic performance is summarized in Supplementary Table 2.

Different thicknesses of PEI-Zn ETL were tested in solar cells with PBDB-T-2F:IT-4F active layer. The devices show comparable performance in the range of 12.3–12.8% when the thickness of PEI-Zn changes from 30–80 nm (Supplementary Fig. 13). Photovoltaic data are summarized in Supplementary Table 3. Electrical conductivity of the PEI-Zn (Zn-to-N, 15:1) was measured using a transmission line method to exclude the influence of contact resistance with a structure in Supplementary Fig. 14a. The electrical conductivity of PEI-Zn (Zn-to-N, 15:1) is 3.4 × 10^−6^ S cm^−1^. Preparation of PEI-Zn includes two stages: (1) Zinc ion is added into the PEI alcohol solution. Based on the suppressed reaction between PEI-Zn solution and IT-4F (Fig. 1c), it can be inferred that the electron lone pairs on the nitrogen interacts with zinc ion; (2) A thermal annealing (110–150 °C) treatment is applied on the PEI-Zn. From the absorption and photoluminescence spectra of the film (Supplementary Fig. 15a, b), there is possible formation of ZnO in the film after the thermal annealing. The long backbone of PEI (molecular weight: 25000) along with the ZnO could be the reason of the film simultaneously displaying good mechanical robustness and a conductivity of 3.4 × 10^−6^ S cm^−1^.

Figure 2d shows the work function of PEI-Zn containing different content of Zn^2+^ measured by Kelvin probe. The reference sample of PEI coated on ITO is about 4.0 eV. The value slightly increases to 4.1 eV after adding Zn^2+^, and the work function is almost independent on the content of Zn^2+^. The PEI-Zn remains the advantage of PEI that can produce low work function and is beneficial to the electron collection in OSCs. X-ray photoelectron spectroscopy (XPS) measurement was performed to confirm the chelation between PEI and Zn^2+^. As shown in Fig. 2e, the binding energy of N 1 s is 398.55 eV for neat PEI. When the Zn^2+^ ions are introduced, the binding energy of N 1 s shifts to blue. As the Zn^2+^ content increases, more N (electron lone pairs) chelated with Zn^2+^. Thus, the shift of binding energy gets larger. The binding energy of Zn 2p also shifts to blue as the content of Zn^2+^ increases (Fig. 2f). These results indicate that electron transfer from N of PEI to Zn^2+^ for the coordination. This electron transfer weakens the electronegativity of amine in PEI, which suppresses the reaction of PEI and IT-4F and improves the solar cell performance. Besides Zn^2+^, we have also tried other metal ions, including Cu^2+^, Ni^2+^, Mn^2+^ and Ag^+^. They can also chelate with amine^39^. However, their work function values (4.60 eV for PEI-Cu, 4.42 eV for PEI-Ni, 4.68 eV for PEI-Mn, and 4.32 eV for PEI-Ag, measured by Kelvin Probe) are not as low as that of PEI-Zn (4.1 eV) for efficient electron collection.

Figure 2g shows the critical strain test on the PEI-Zn and ZnO reference films. The films were deposited on 175-µm thick polyethersulfone (PES) substrates. The individual films on PES were bended at different bending radius and their resistance was measured before and after bending. For ZnO film, the resistance starts to increase when the bending radius (*r*) is less than 5 mm. For PEI-Zn film, the resistance keeps unchanged until *r* is as low as 2.2 mm. According to the bending strain equation (*ε* = *H*_s_/(2*r*), where *H*_s_ is the thickness of the PES substrate), the maximum bending strain of PEI-Zn (3.98%) is over twice as high as that of ZnO (1.75%). Figure 2h, i are the scanning electron microscope (SEM) images of PEI-Zn and ZnO films on PES substrates after bending for 500 times with a bending radius of 4 mm. No cracks were observed on the SEM image of PEI-Zn film after the continuous bending (Fig. 2h). On the contrary, cracks can be observed for ZnO film after the same bending conditions (Fig. 2i).

### Mechanical robustness of the ultraflexible OSCs

**Fig. 3:**
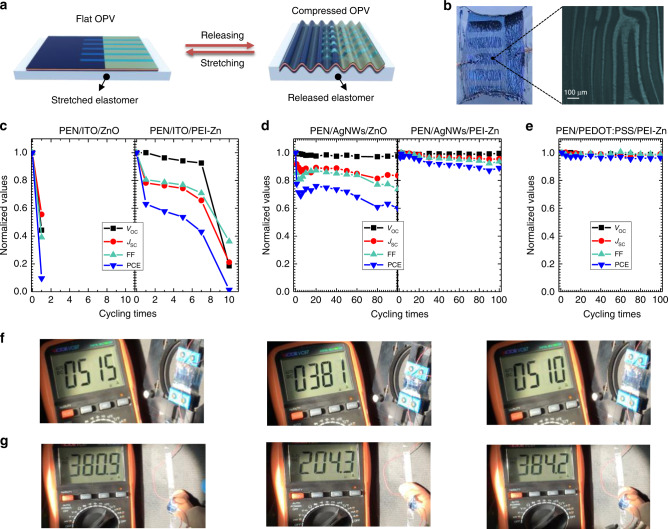
Extreme mechanical flexibility. **a** Illustration of the ultrathin OSCs attached onto the 300% pre-stretched elastomer (VHB) to demonstrate the extreme flexibility; the cells are at flat and compressed states by stretching and releasing the elastomer. When the elastomer is stretched to the pre-stretched state, the ultrathin cell becomes flat. When the elastomer is released, the cell is at the compressed state. **b** Picture of ultrathin cells on a released elastomer with a 45% compression. The right is an image of an compressed cell under an optical microscope; Evolution of photovoltaic parameters after different compressed-flat cycles of ultraflexible of cells with different electrode/ETL: **c** with PEN/ITO/ZnO and PEN/ITO/PEI-Zn; **d** with PEN/AgNWs/ZnO and PEN/AgNWs/PEI-Zn; **e** with PEN/PEDOT:PSS/PEI-Zn; Pictures captured from the supplementary videos to demonstrate the current recovery during the cycling: **f** cell on a stretching-releasing apparatus; **g** cell attached on a finger joint.

To test mechanical robustness of the ultraflexible cell, we transferred the cell to a pre-strained elastomer, VHB, with 300% of the initial length. The cell is at flat or compressed state by stretching and releasing the elastomer, as shown in Fig. 3a. When the elastomer is at the pre-strained state, the ultrathin cell is flat. When the elastomer is released, the cell is at the compressed state. The cycling was performed on the apparatus shown in Supplementary Fig 16. At the compressed state, wrinkled stripes can be observed perpendicular to the direction of strained force. The radius of the wrinkles reaches lower than 20 μm (Fig. 3b). When the elastomer is stretched, the wrinkles become sparser and finally disappear at the flat state (Supplementary Fig. 17). The deformation is quantified as *ΔL*/*L*_0_, where *L*_0_ is the length of the cell when the cell is at flat state (defined as the initial state), and *ΔL* is the changed length of the cell when elastomer is released. Supplementary Fig. 18 shows the current-voltage (*I*-*V*) characteristics of the cell at different deformation states. The *V*_OC_ and FF remain high almost without change (*V*_OC_ = 0.83 V and FF = 0.70) when the cell is at compression ratio up to 45% (*ΔL*/*L*_0_).

To further test the mechanical robustness of the cells, continuous deformation cycling test (45% compression and flat) has been performed. Cells with different electrodes (ITO, AgNWs and PEDOT:PSS) and different ETL (PEI-Zn and ZnO) have been fabricated. Ultraflexible OSCs with PEI-Zn show higher PCE for all tested different structures, compared to ZnO (Supplementary Fig. 19). Figure 3c shows the ultrathin cells with ITO electrodes degrade very rapidly during the cycling test. The performance is reduced to about 10% of its initial PCE after one-time cycling. When ZnO ETL is replaced by PEI-Zn, the cell shows improved mechanical property. The PCE remains 60 and 40% of initial PCE after one-time and seven times cycling. But, the overall mechanical robustness is still poor, which is due to the brittle properties of ITO electrode^40^. It should be note that there is no barrier layer here (such as parylene) coated on top of the ITO-based ultrathin cells. The barrier layer enables the ITO embedding at a neutral strain position and suffering less strain, and therefore better mechanical robustness was achieved in the previous work^21,25^. The ITO here suffers higher stain and the test condition is harsher. Figure 3d shows the mechanical properties of the cells with AgNWs electrodes. The PEI-Zn ETL-based cells also deliver better mechanical robustness than the ZnO ETL-based cell. After the first cycle, the efficiency becomes 77% of initial PCE for the ZnO ETL-based cell, while 97% of its initial PCE for the PEI-Zn ETL-based cell. After 100 deformation cycling, the PEI-Zn ETL-based cell still has 89% of its initial PCE. When the transparent electrode is changed to PEDOT:PSS, PCE of the cell (with PEI-Zn ETL) is almost unchanged during the 100 continuous deformation cycles (Fig. 3e). These results suggest the PEI-Zn can deliver good mechanical properties for the ultraflexible solar cells even not at a neutral strain position. Note that mechanical flexibility of cells with PEDOT:PSS/ZnO is not studied for comparison because the device showed poor performance, which is associated the chemical reduction of PEDOT:PSS by sol-gel ZnO. The color of PEDOT:PSS film changed from light blue to dark blue after spin-coating ZnO with thermal annealing (Supplementary Fig. 20). Two peaks at around 650 nm and 950 nm are observed in the absorption spectrum of PEDOT:PSS/ZnO, which are assigned to the polaron-induced absorption and neutralized absorption of PEDOT^41^. Sheet resistance of PEDOT:PSS film increases from 110 ohm sq^−1^ to around 430 ohm sq^−1^ after the ZnO coating. The increased absorbance and sheet resistance result in poor performance.

Then, we attached a cell on a finger joint to demonstrate its functioning under compressing-releasing cycles. Under a solar simulator, the current of the stretchable solar cells changes periodically with the finger bending and spreading. Supplementary Movies 1 and 2 show the compressing-releasing cycling of the ultrathin cell on the stretching-releasing apparatus and the bending-spreading finger, separately. The current of the cell under the solar simulator could change back and forth during the cycling recorded by the multimeter. Fig. 3f, g show the pictures captured from the supporting videos that show the current recovers during the cycling.

## Discussion

We have presented a low–work function interlayer of Zn^2+^ chelated polyethylenimine (PEI-Zn) that can endure high mechanical bending strain, and simultaneously has good chemical compatibility with the efficient nonfullerene active layers. Using the PEI-Zn interlayer, ultraflexible solar cells with PCE up to 15% have been demonstrated. Furthermore, the ultraflexible solar cells show nearly unchanged PCE during 100 continuous compression-flat deformation cycles with a compression ratio of 45%. The PEI-Zn layer is solution-processed and works efficiently with the printable flexible transparent electrodes of metal nanowires (such as AgNWs) and conducting polymer PEDOT:PSS. Cells with doctor-bladed AgNWs/PEI-Zn electrodes show comparable performance to spin-coated electrodes (Supplementary Fig. 21). The PEI-Zn can be very promising in printed electronics and flexible electronics.

## Methods

### Materials

PBDB-T-2F and IT-4F were purchased from Solarmer Materials Inc. Y6 is purchased eFlexPV Technology (Shenzhen) Co., Ltd. Zinc acetate dihydrate, methoxyethanol, PEIE (80% ethoxylated solution, 37 wt.% in H_2_O) and polyethylenimine (PEI, Mw 25000) for PEI-Zn precursor solutions were purchased from Sigma-Aldrich. PEDOT:PSS aqueous solution (Clevios PH1000) was purchased from Xi’an Polymer Light Technology Corp. Ethylene glycol, and 1.3-μm-thick PEN films were purchased from Sigma-Aldrich. The sol-gel ZnO solution was prepared by dissolving 0.1 g Zinc acetate dihydrate and 0.028 g ethanolamine (Sigma-Aldrich) in 1 mL methoxyethanol. To prepare PEI-Zn precursors, 0.1 wt.% PEI or 0.5 wt.% PEIE (plus water) was dissolved in methoxyethanol. Then, different amount of zinc acetate dihydrate or zinc acetylacetonate was added into the PEI or PEIE solution, and stirred for 3 h before coating. For the optimized Zn-to-N mole ratio of 15:1, 0.075 g zinc acetate dihydrate was added to the 1 mL 0.1 wt.% PEI or 0.5 wt.% PEIE (plus water) solution and stirred until the solution became clear. Adding a small amout (10 μL) of water faciliates the dissolving of Zinc acetate dihydate or Zinc acetylacetonate hydrate in PEI or PEIE solution. 

### Device fabrication

The ultrathin PEN substrates were firstly cut into the size of around 2.3 × 2.3 cm^2^ and adhered onto PDMS-coated glass substrates. PDMS films was prepared by a mixture of base and crosslinker (10:1, weight ratio, PP2-OE41, Gelest Inc.) and cured on a hot plate in air at 80 °C for 40 min. The bottom electrode of PEDOT:PSS was prepared by spin-coating the PH1000 consisting of 5 wt.% ethylene glycol and 0.1 wt.% superwet-340 surfactant and patterned through surface energy tuning via plasma treatment. As for AgNWs electrode, AgNWs solution of 2 mg mL^−1^ was spin-coated on substrates at a speed of 2000 rpm, following an annealing at 200 °C for 10 min. PEDOT:PSS and AgNWs electrodes were patterned under a fetosecond laser (Hongtuo, Wuhan). Next, PEI-Zn precursor solution was spin-coated at 3000 rpm for 40 s, following a thermal annealing at 150 °C for 10 min. The PEI-Zn was spin-coated on PEDOT:PSS electrodes and AgNWs electrodes (PEI-Zn was spin-coated twice on AgNWs to flatten the surface). When the mole ratio of Zn-to-N was smaller than 3:1, the films were thinner than 10 nm. The thicknesses were 12, 19, and 25 nm for the films prepared from precursors with mole ratios (Zn-to-N) of 4:1, 10:1 and 15:1, respectively. Then, the substrates were transferred to the N_2_-filled glovebox. PBDB-T-2F:IT-4F (1:1, weight ratio) in a 20 mg mL^−1^ chlorobenzene:DIO (99.5:0.5, volume ratio) solution was spin-coated at 1500 rpm for 60 s. PBDB-T-2F:Y6 (1:1.2, weight ratio) in a 16 mg mL^−1^ chloroform:chloronaphthalene (CN) (99.5:0.5, volume ratio) solution was spin-coated at 2500 rpm for 60 s. After a thermal annealing at 100 °C for 10 min, the samples were transferred into the vacuum evaporation system (Mini-spectros, Kurt J. Lesker). 7-nm-thick MoO_3_ and 100-nm-thick Ag were evaporated with a metal shadow mask under high vacuum (<5 × 10^−7^ Torr). The area of single cell is 4.1 mm^2^. Before measurement, the devices were annealed at 100 °C for 10 min in the N_2_-filled glove box. Stretching-releasing apparatus and operational procedure were shown in Supplementary Fig. 16 and Supplementary Movie 1.

### Characterization of film and devices

Current density-voltage (*J*-*V*) characteristics of the cells were measured using a Keithley 2400 SourceMeter. The cells were illuminated through an aperture area of 4.095 mm^2^ from a 100 mW cm^−2^ AM1.5 solar simulator (Newport, ORIEL, Sol3A, 450 W xenon lamp). The long-term photostability of devices was tested with an ultraviolet filter the cut off the light illumination below 400 nm. The area of the aperture is confirmed by the National Institute of Metrology (NIM, Beijing). The measurement was performed in a N_2_-fillerd glove box at the temperature of 25 °C. The light source is a solar simulator (Newport, Sol3A) with a 450 watt xenon lamp (Newport). Light intensity of the light source was calibrated using a NIST-certified monocrystalline Si solar cell (Newport 532 ISO1599). There is almost no variation of the performance with or without aperture. The cells were measured with a step voltage of 0.04 V and a dwell time of 0.2 s for every point. Absorbance and transmittance of films was characterized using a UV-vis-NIR Spectrophotometer (UV3600, Shimadzu). Reflectance of the cells with different deformation ratios was also conducted by the UV-vis-NIR Spectrophotometer with an integrating sphere. The valence states of Zn and N were measured by X-ray photoelectron spectrometer (XPS) (Kratos Analytical). The SEM images of PEI-Zn (Zn-to-N ratio of 15:1) and ZnO after bending for 500 times were conducted by FEI Nova Nano-SEM 450. The work function was carried out by Kelvin probe (KP-020, KP Technology). The photographic images of the ultrathin devices on a pre-strained elastomeric tape under different compression were taken by using optical microscope (Nikon ECLIPSE LV150). The EQE test was performed by a standard system using a 150 W xenon lamp (Newport) fitted with a monochromator (Cornerstone 74004) as a monochromatic light source. The NMR spectra were collected on a Bruker Ascend 400 MHZ NMR spectrometer using deuterated dichloromethane as the solvent and tetramethylsilane as the internal standard. The liquid chromatography–mass spectrometry measurement was performed on an Agilent 1100 LC-MS Trap XCT System (Agilent Technologies, USA).

### Reporting summary

Further information on research design is available in the Nature Research Reporting Summary linked to this article.

## Supplementary information

Supplementary Information

Description of Additional Supplementary Files

Supplementary Movie 1

Supplementary Movie 2

Reporting Summary

Solar Cells Reporting Summary

## Data Availability

The data that support the findings of this study are available from the corresponding author upon reasonable request. Source data are provided with this paper.

## Source data

Source Data
