# Supplementary material for: Robust metal ion-chelated polymer interfacial layer for ultraflexible non-fullerene organic solar cells
Source: Nat Commun. 2020 Sep 9;11:4508. doi: 10.1038/s41467-020-18373-0 (PMC7481191; doi:10.1038/s41467-020-18373-0)
Supplement: Supplementary file 1 — Supplementary Information [file 41467_2020_18373_MOESM1_ESM.pdf]

Supplementary Information for

Robust metal ion-chelated polymer interfacial layer for ultraflexible non-fullerene organic solar cells

Qin et al.

Supplementary Figures

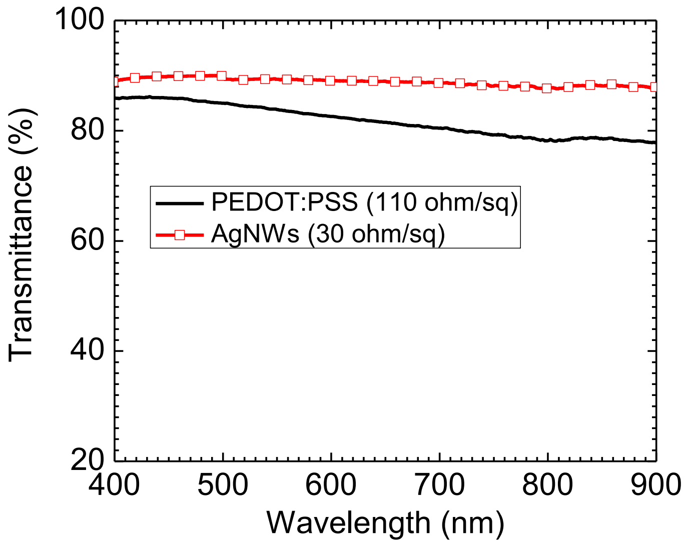

Supplementary Figure 1 Transmittance spectra of glass/PEDOT:PSS and glass/AgNWs electrodes, measured with an integrating sphere.

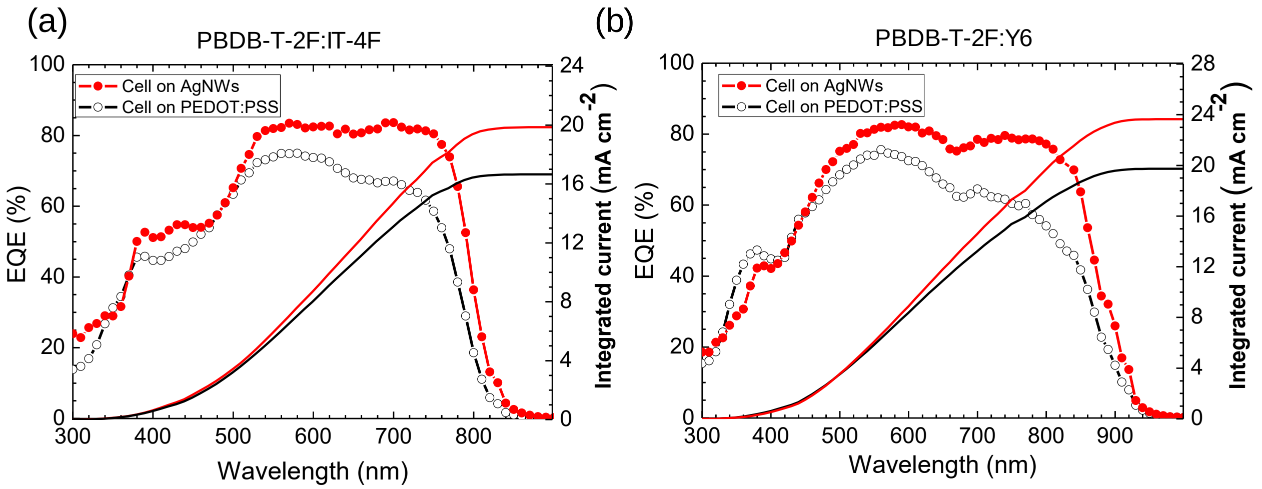

Supplementary Figure 2 EQE spectra of ultrathin cells on PEDOT:PSS and AgNWs electrodes: (a) with PBDB-T-2F:IT-4F active layer; (b) with PBDB-T-2F:Y6 active layer.

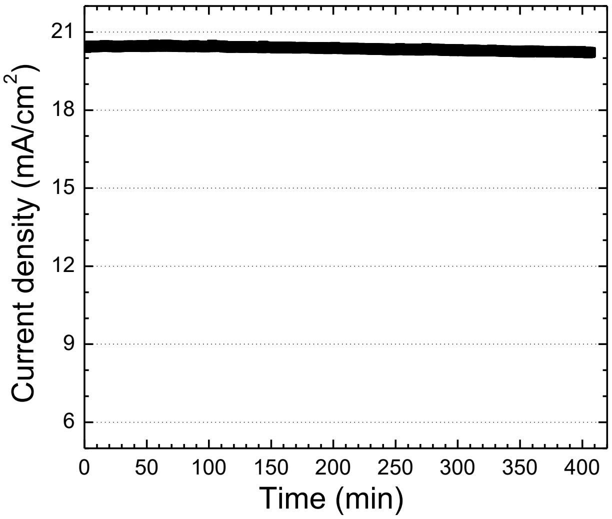

Supplementary Figure 3 Stabilized photocurrent density output of an ultrathin cell (PEN/AgNWs/PEI-Zn/ PBDB-T-2F:IT-4F/MoO_3/Ag) under continuous one sun illumination in a N_2-filled glove box. (light below 400 nm was filtered). The applied voltage is at V_{mpp} of 0.64 V.

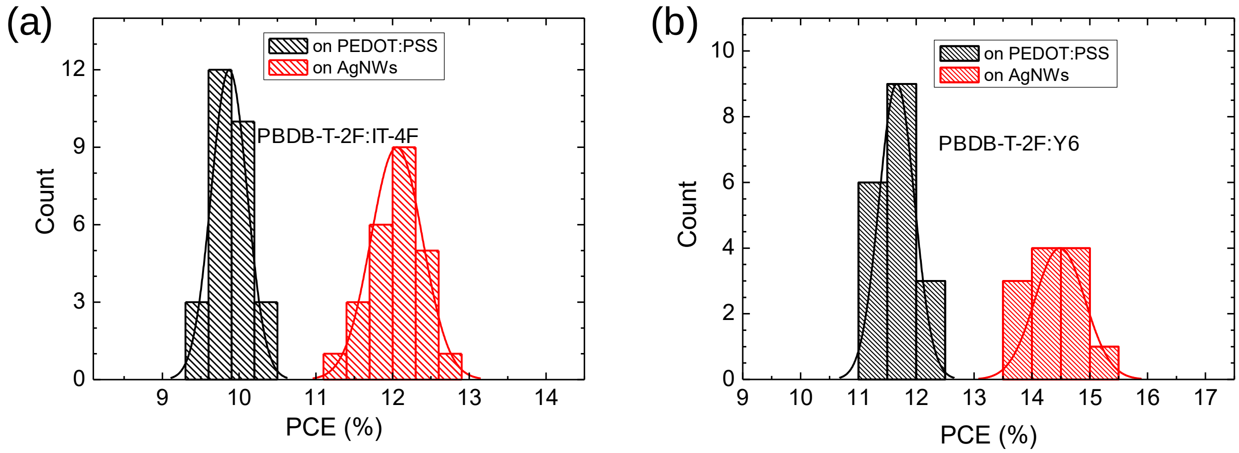

Supplementary Figure 4 Histogram of PCE distribution of the four different types of ultraflexible OSCs: (a) with PEDOT:PSS/PBDB-T-2F:IT-4F and AgNWs/PBDB-T-2F:IT-4F; (b) PEDOT:PSS/PBDB-T-2F:Y6 and AgNWs/PBDB-T-2F:Y6.

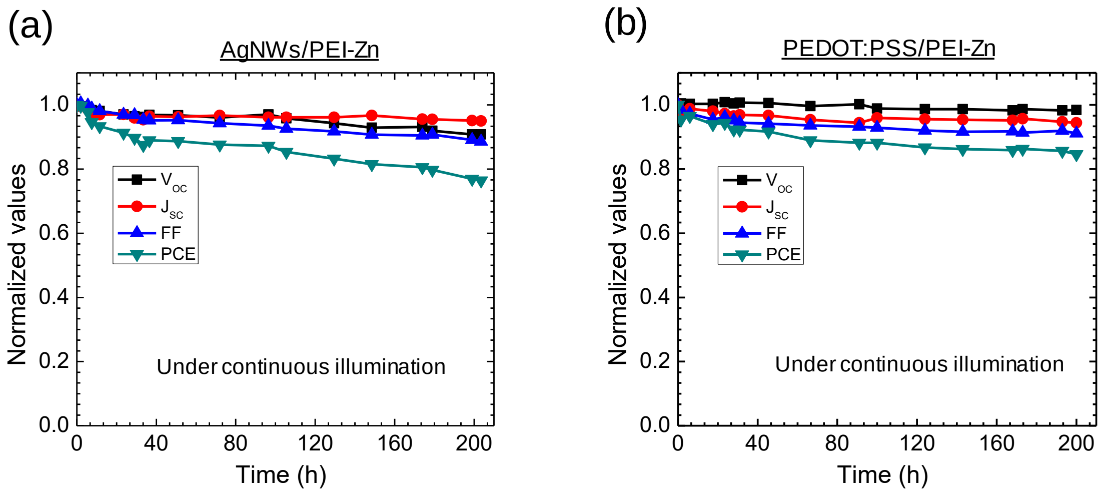

Supplementary Figure 5 Normalized photovoltaic parameter change of the cells (with 100-nm Al_2O_3 encapsulation) exposed to 100 mW cm^{-2} illumination (light below 400 nm was filtered) in air for different time. The cells have different electrodes: (a) AgNWs/PEI-Zn; (b) PEDOT:PSS/PEI-Zn.

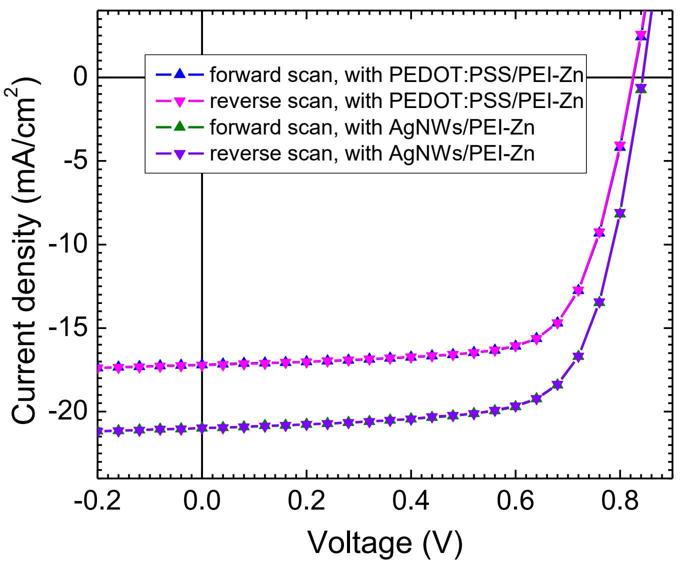

Supplementary Figure 6 *J-V* characteristics of the ultraflexible cells on PEDOT:PSS and AgNWs electrodes in both forward and backward scan directions with PBDB-T-2F:IT-4F active layer.

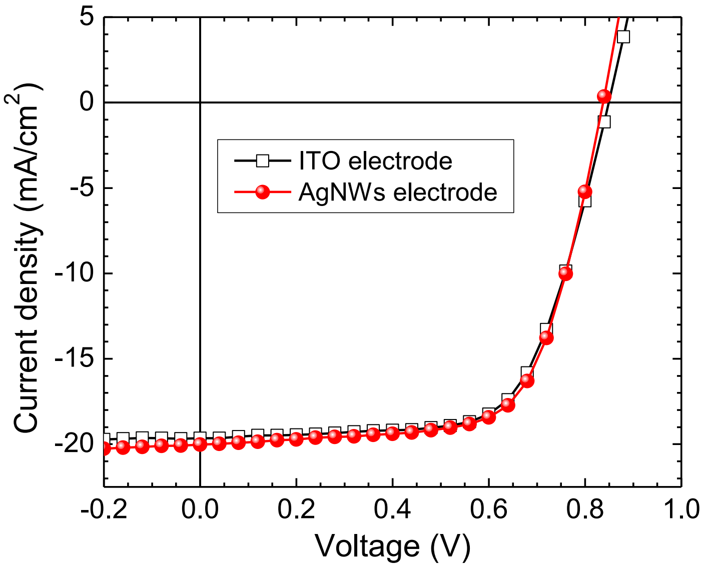

Supplementary Figure 7 *J-V* characteristics of large-area (1 cm^2) ultrathin solar cells on PEN/AgNWs/PEI-Zn with PBDB-T-2F:IT-4F active layer. The cell on glass/ITO/PEI-Zn electrode with 1-cm^2 area is also fabricated as a reference.

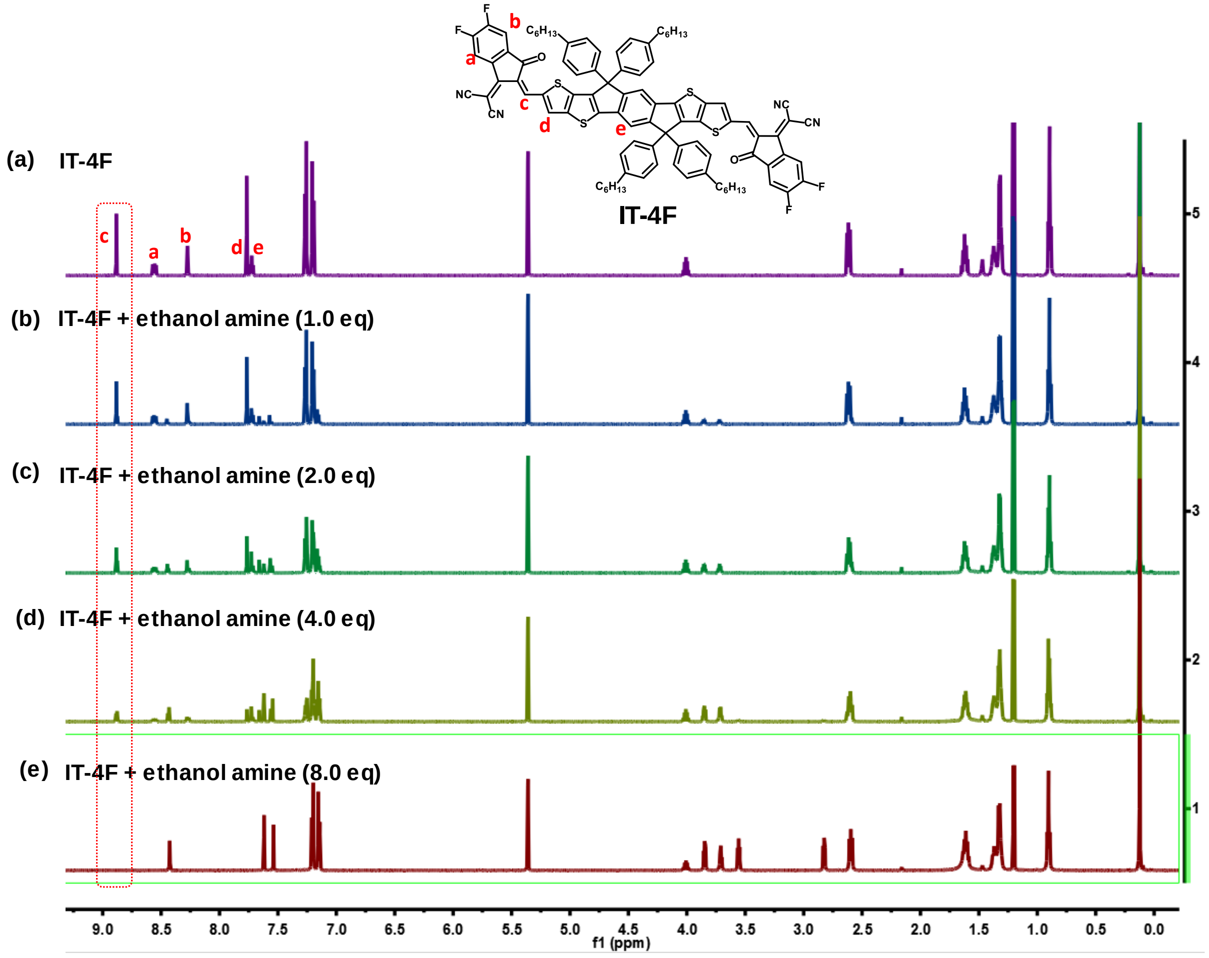

Supplementary Figure 8 ^{1}H NMR spectra of pristine IT-4F and IT-4F after adding different equivalent fractions of ethanolamine. The reagent is CD_2Cl_2. The marked dash box is to show the intensity change of H at the position of marked "c" in the chemical structure.

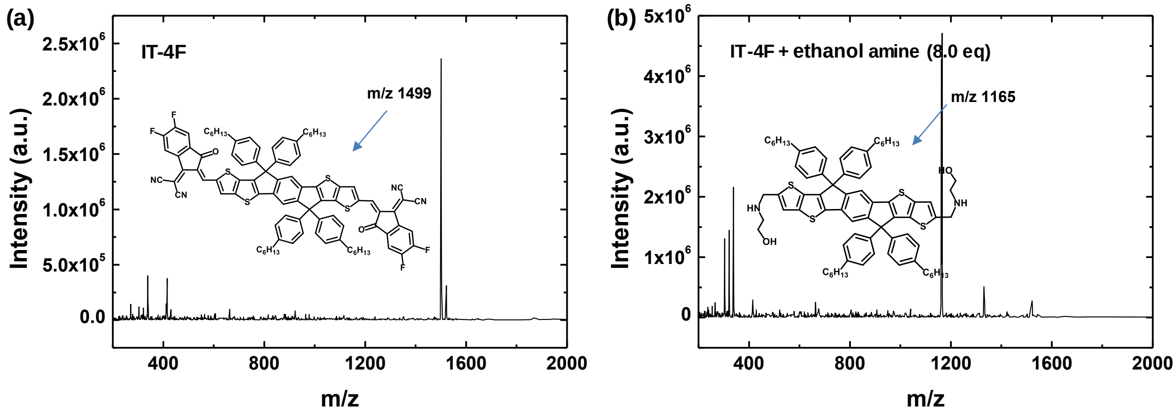

Supplementary Figure 9 Mass spectra: (a) pristine IT-4F; (b) IT-4F after adding 8.0 eq ethanolamine. The pristine IT-4F delivers an m/z of 1499. After introducing 8.0 eq ethanol amine, the emerging fragment at m/z of 1165 attributes to the addition of ethanol amine to both C=C linkage moieties and then the loss of terminal acceptor moieties.

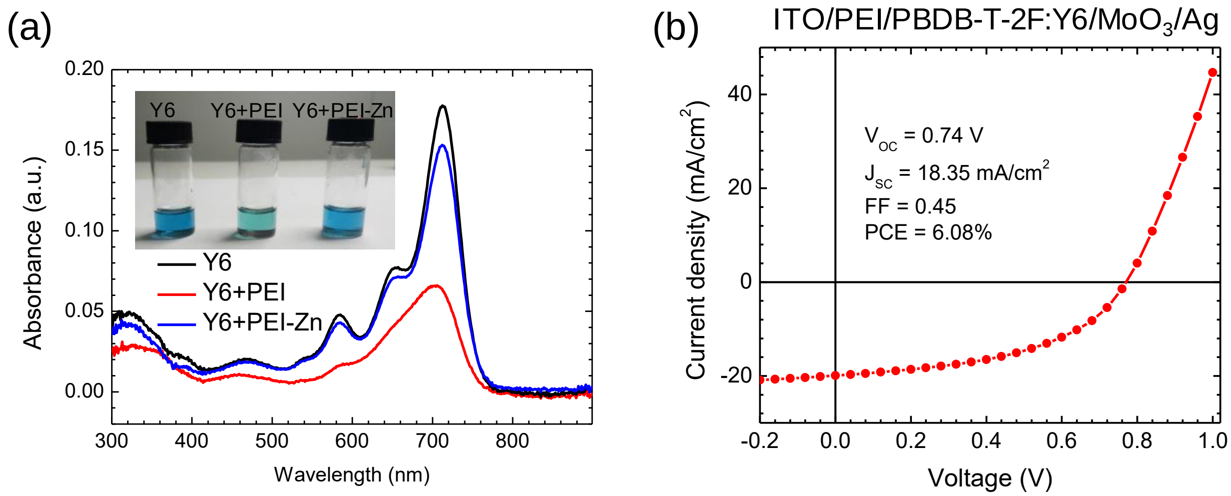

Supplementary Figure 10 (a) Absorption spectra of three solutions: Y6, Y6 mixed with PEI, and Y6 mixed with PEI-Zn solution. The inset shows the pictures of the three solutions. 100 μL 0.1 wt.% PEI was added to 1 mL 0.02 mg mL^{-1} Y6 solution to study the reaction between them. For PEI-Zn test, additional $Zn(CH_3COO)_2$ was added into the PEI solution (Zn-to-N 15:1) before mixing with Y6. (b) *J*-*V* characteristics of solar cells with the structure of ITO/PEI/PBDB-T-2F:Y6/MoO_3/Ag.

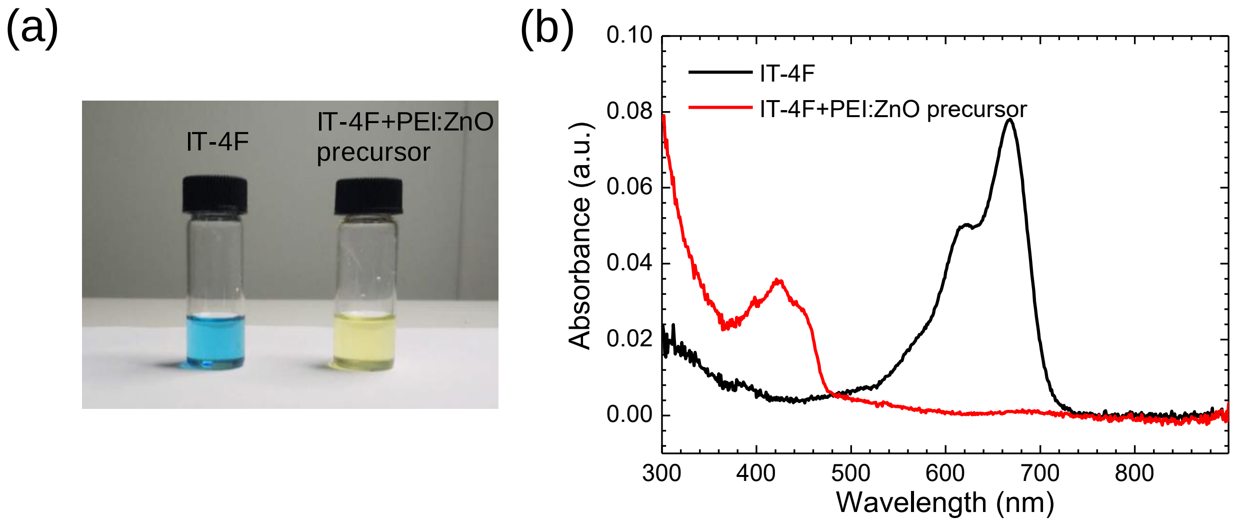

Supplementary Figure 11 (a) Photographs of IT-4F and IT-4F mixed with PEI:ZnO precursor after 12 hours. 100 μL PEI:ZnO precursor (0.1 wt.% PEI is dissolved in sol-gel ZnO precursor) was added to 1 mL 0.02 mg/mL IT-4F solution to study the reaction. (b) Absorption spectra of two solutions: IT-4F and IT-4F mixed with PEI:ZnO precursor after 12 hours.

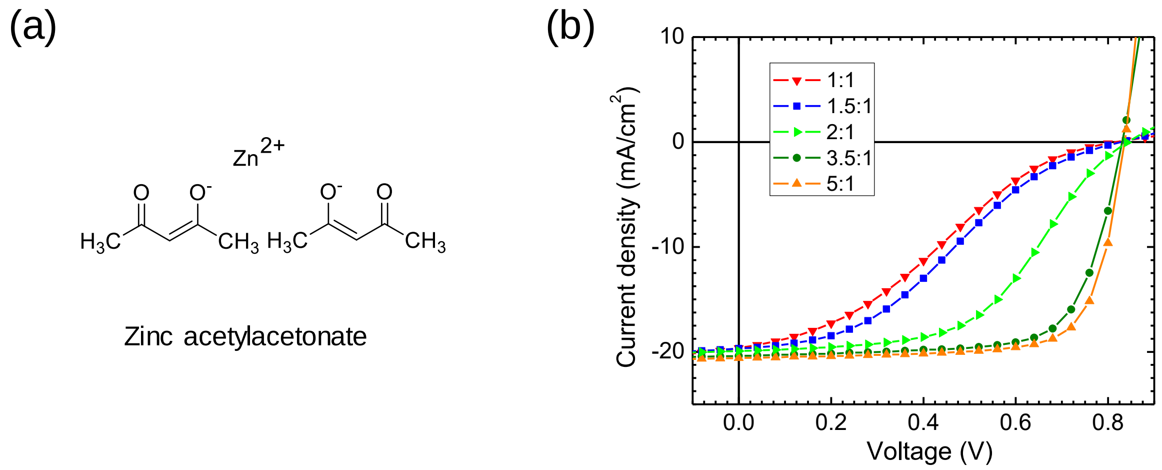

Supplementary Figure 12 (a) Chemical structure of the zinc acetylacetonate; (b) *J-V* characteristics of the cells where the PEI-Zn are prepared by adding different ratios of zinc acetylacetonate into the PEI solutions. Zinc acetylacetonate provides the Zn^{2+} source. Zn-to-N mole ratio is shown in the legend.

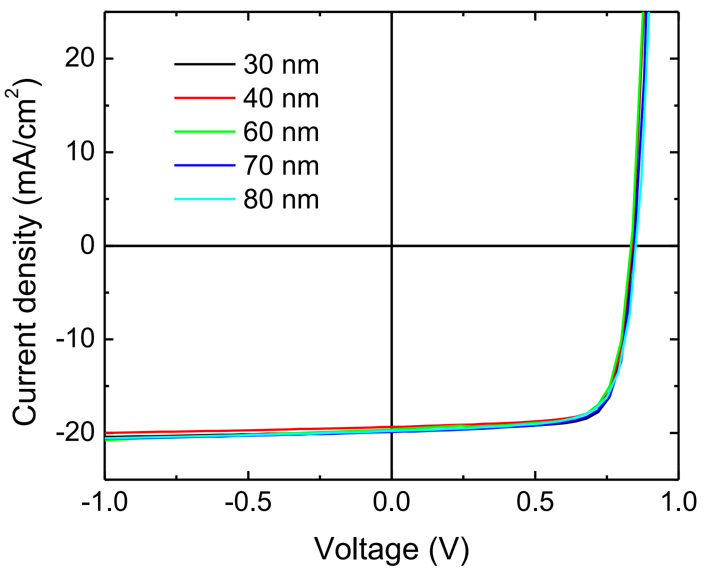

Supplementary Figure 13 *J*-*V* characteristics of solar cells with different thickness of PEI-Zn. The devices have a structure of ITO/ETL/PBDB-T-2F:IT-4F/MoO_3/Ag.

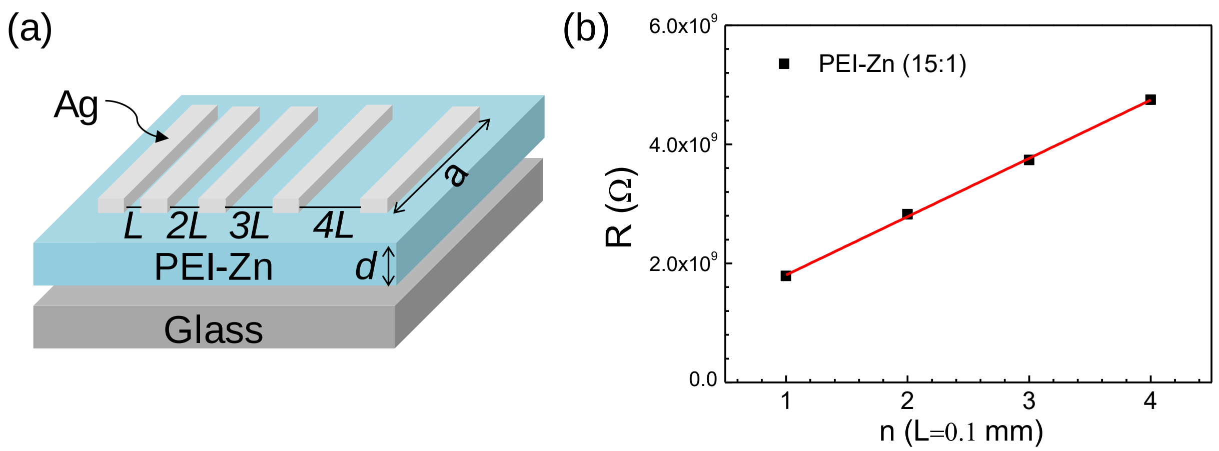

Supplementary Figure 14 (a) Structure of device for conductivity measurement of the PEI-Zn films. (b) Resistances as a function of length. Resistances of PEI-Zn with different length are measured to exclude the influence of contact resistance. Slope of the line in **Supplementary Figure 14b** is the resistance of PEI-Zn with a length of L ($R_L = \frac{\Delta R}{\Delta n}$). The conductivity (σ) can be evaluated by the following equation: $\sigma = \frac{L}{adR_L}$, where L and a are the length and width of measured PEI-Zn film; d denotes the thickness. The thickness of measured PEI-Zn is 30 nm. The L and a are 0.1 mm and 10 mm.

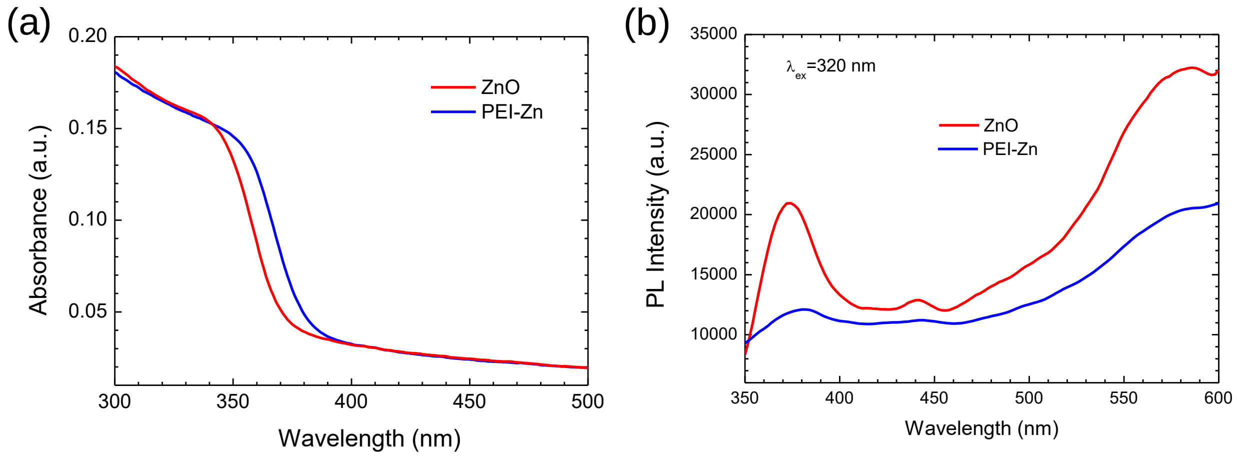

Supplementary Figure 15 (a) UV-Vis spectra of ZnO and PEI-Zn films. (b) Photoluminescence spectra of ZnO and PEI-Zn films.

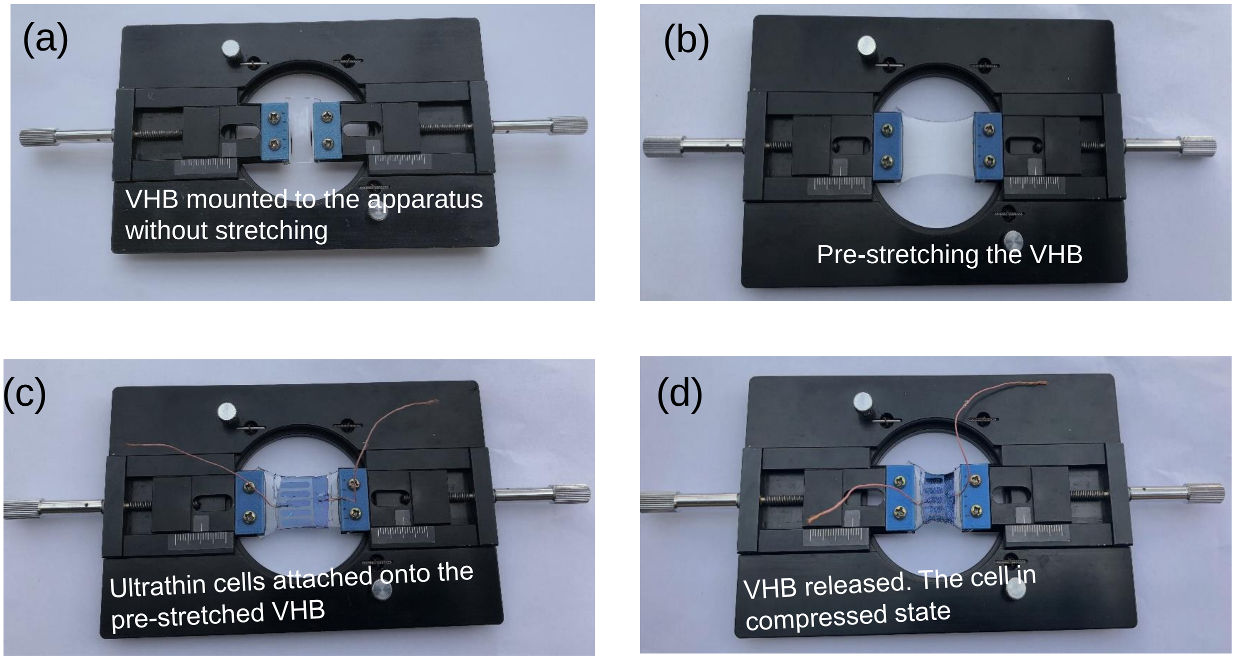

Supplementary Figure 16 Pictures of the ultrathin cells attached onto the stretching-releasing apparatus. (a) VHB elastomer mounted to the apparatus without stretching; (b) The VHB elastomer is pre-stretched; (c) Ultrathin cells are attached onto the pre-stretched VHB. The cells are at flat state. Two wires contact anode and cathode of the cell, separately. (d) The VHB is released. The cell is at the compressed state. The compressed state is defined as the initial state for the stretching test and measurement.

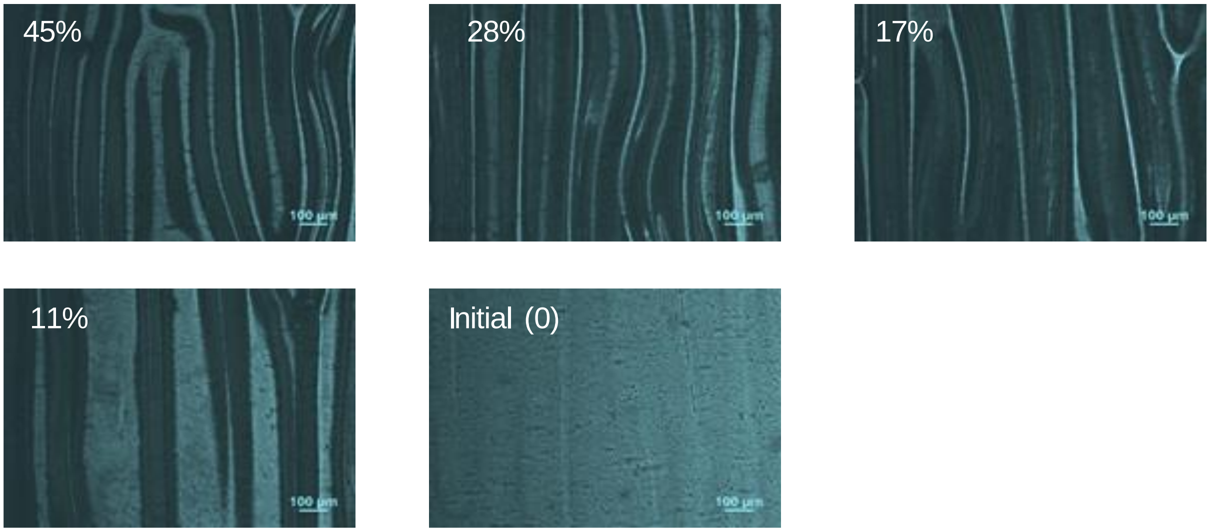

Supplementary Figure 17 Photographic images of a cell at different compression ratios under an optical microscope.

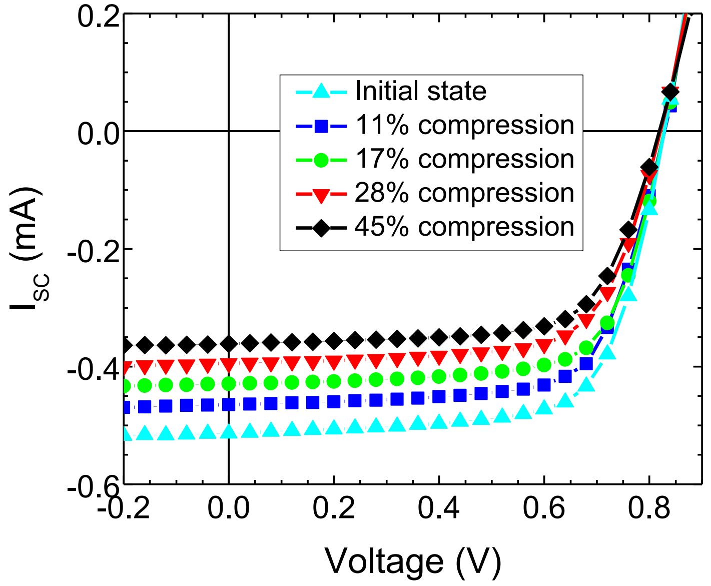

Supplementary Figure 18 Current-voltage (*I-V*) characteristics of the cell at different compression states. The compression ratio is quantified as $\Delta L/L_0$, where L_0 is the length of the cell at flat state (defined as the initial state), and ΔL is the changed length of the cell when elastomer is released.

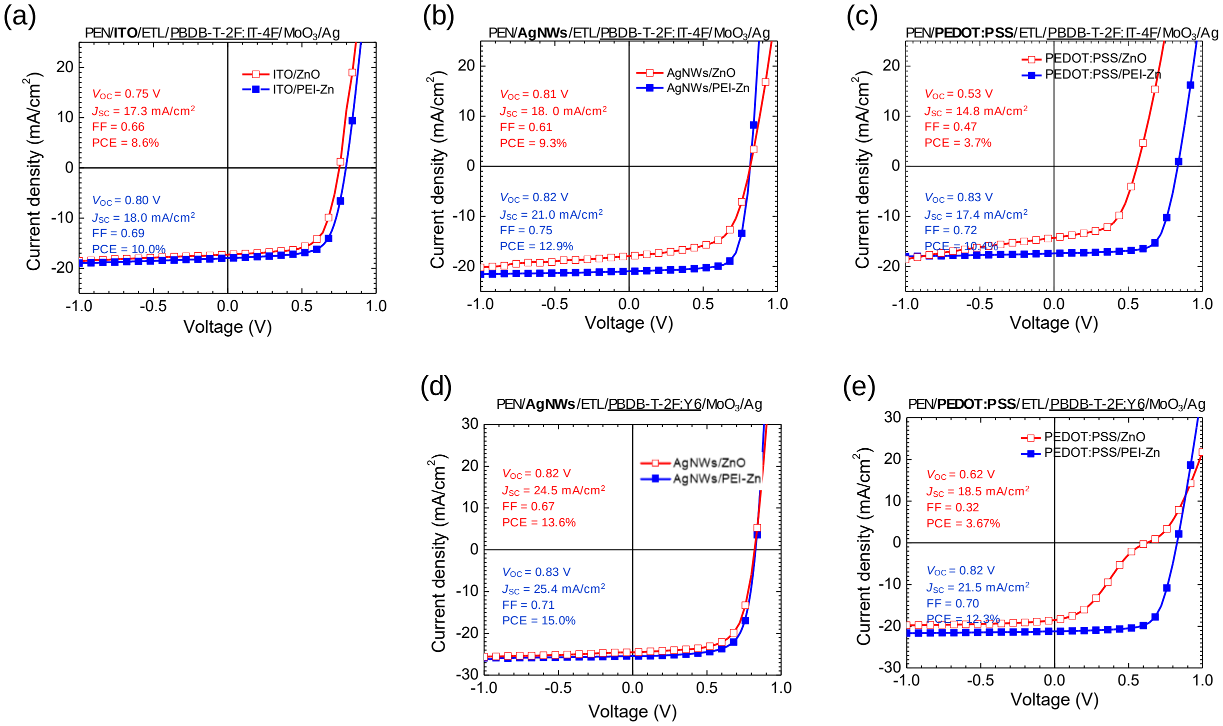

Supplementary Figure 19 *J-V* characteristics of the ultrathin cells fabricated with different electrodes: (a-c) on ITO, AgNWs and PEDOT:PSS electrodes, respectively. The active layer is PBDB-T-2F:IT-4F. (d-e) on AgNWs and PEDOT:PSS electrodes, respectively. The active layer is PBDB-T-2F:Y6.

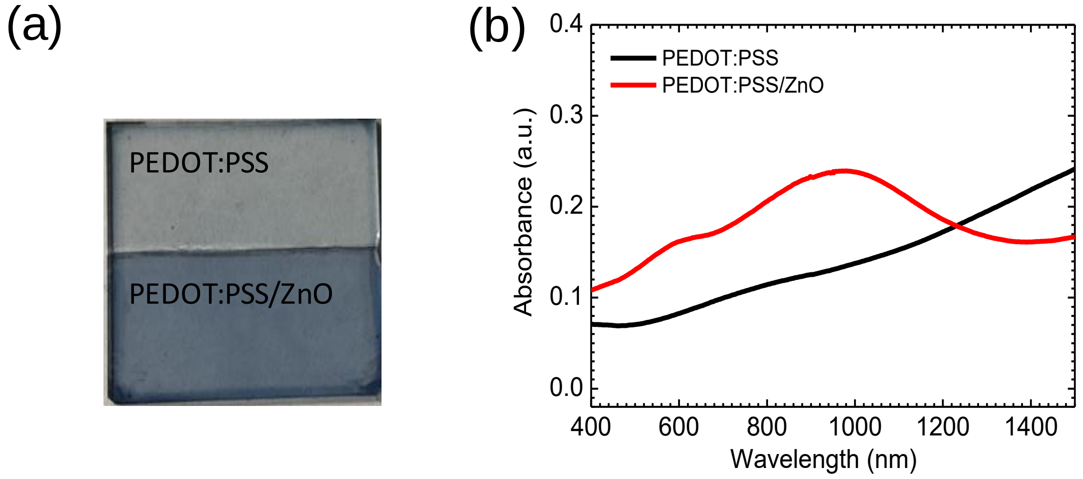

Supplementary Figure 20 (a) Pictures of the PEDOT:PSS films with and without sol-gel ZnO coating; (b) Absorbance spectra of the PEDOT:PSS films with and without sol-gel ZnO coating.

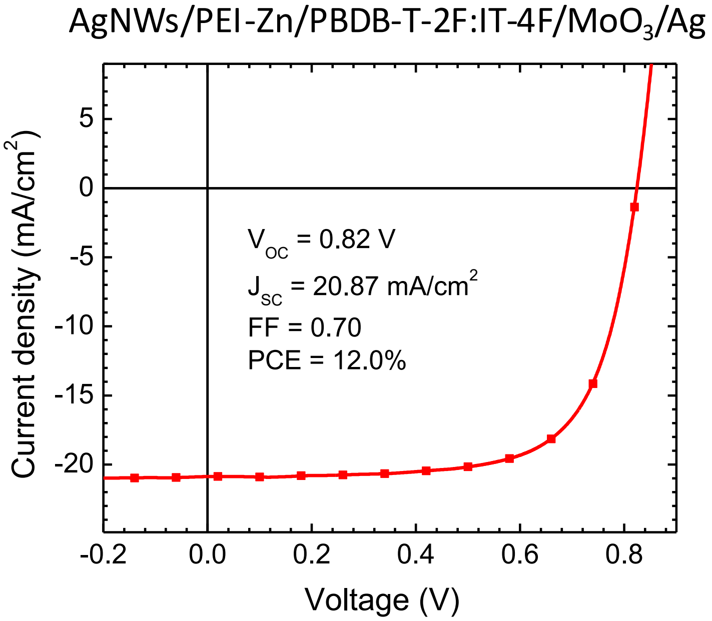

Supplementary Figure 21 *J-V* characteristics of the cells with both AgNWs and PEI-Zn fabricated by doctor blading.

Supplementary Tables

Supplementary Table 1 Photovoltaic performance of the inverted solar cells with PEI-Zn whose zinc source is from ***zinc acetate***. The device structure is glass/ITO/ PEI-Zn/PBDB-T-2F:IT-4F/MoO_3/Ag.

ETL	V_{OC} (V)	J_{SC} (mA cm^{-2})	FF	PCE (%)
0:1	0.76	18.92	0.30	4.31
2:1	0.81	20.10	0.34	5.52
3:1	0.82	19.88	0.57	9.25
4:1	0.82	20.72	0.73	12.41
15:1	0.84	20.81	0.76	13.29
20:1	0.10	10.83	0.25	0.28

Supplementary Table 2 Photovoltaic performance of the inverted solar cells with PEI-Zn whose zinc source is from ***zinc acetylacetonate***. The device structure is glass/ITO/ PEI-Zn/PBDB-T-2F:IT-4F/MoO_3/Ag.

ETL	V_{OC} (V)	J_{SC} (mA cm^{-2})	FF	PCE (%)
1:1	0.82	19.64	0.29	4.67
1.5:1	0.82	19.76	0.28	4.54
2:1	0.83	19.64	0.48	7.82
3.5:1	0.83	20.39	0.71	12.02
5:1	0.84	20.60	0.74	12.80

Supplementary Table 3 Photovoltaic performance of the inverted solar cells with different thickness of PEI-Zn. The device structure is glass/ITO/**ETL**/PBDB-T-2F:IT-4F/MoO_3/Ag.

Thickness (nm)	V_{OC} (V)	J_{SC} (mA cm^{-2})	FF	PCE (%)
30	0.84	19.8	0.77	12.8
40	0.84	19.4	0.76	12.4
60	0.83	19.6	0.75	12.2
70	0.84	19.9	0.75	12.5
80	0.84	19.8	0.74	12.3
