# Supplementary material for: Robust metal ion-chelated polymer interfacial layer for ultraflexible non-fullerene organic solar cells
Source: Nat Commun. 2020 Sep 9;11:4508. doi: 10.1038/s41467-020-18373-0 (PMC7481191; doi:10.1038/s41467-020-18373-0)
Supplement: Supplementary file 2 — Description of Additional Supplementary Files [file 41467_2020_18373_MOESM2_ESM.pdf]

Description of Additional Supplementary Files

Supplementary Movie 1 demonstrates the compressing-releasing cycling of an ultraflexible cell under o perational condition on an apparatus.

Supplementary Movie 2 demonstrates the compressing-releasing cycling of an ultraflexible cell under operational condition on a finger joint.
