# Supplementary material for: Robust metal ion-chelated polymer interfacial layer for ultraflexible non-fullerene organic solar cells
Source: Nat Commun. 2020 Sep 9;11:4508. doi: 10.1038/s41467-020-18373-0 (PMC7481191; doi:10.1038/s41467-020-18373-0)
Supplement: Supplementary file 6 — Solar Cells Reporting Summary [file 41467_2020_18373_MOESM6_ESM.pdf]

nature research

Corresponding author(s): Yinhua Zhou

Solar Cells Reporting Summary

Nature Research wishes to improve the reproducibility of the work that we publish. This form is intended for publication with all accepted papers reporting the characterization of photovoltaic devices and provides structure for consistency and transparency in reporting. Some list items might not apply to an individual manuscript, but all fields must be completed for clarity.

For further information on Nature Research policies, including our data availability policy, see Authors & Referees.

ü Experimental design

Please check: are the following details reported in the manuscript?

1. Dimensions

Area of the tested solar cells	[x] Yes [ ] No	Described in "Methods" section. The area of 4.1 mm^2.
Method used to determine the device area	[x] Yes [ ] No	Described in "Methods" section. Aperture of 4.095 mm^2 used to determine the area. The area is confirmed by the National Institute of Metrology (NIM, Beijing).

2. Current-voltage characterization

Current density-voltage (J-V) plots in both forward and backward direction	[x] Yes [ ] No	Reported in Supplementary Figure 6.
Voltage scan conditions<br>*For instance: scan direction, speed, dwell times*	[x] Yes [ ] No	Described in "Methods" section. Cells are measured in both forward and backward directions with a step voltage of 0.04 V. The dwell time is 0.2 s for every point.
Test environment<br>*For instance: characterization temperature, in air or in glove box*	[x] Yes [ ] No	Described in "Methods" section. The J-V characteristics were conducted in glove box at the temperature of 25 °C.
Protocol for preconditioning of the device before its characterization	[ ] Yes [x] No	No preconditioning of the device before its characterization.
Stability of the J-V characteristic<br>*Verified with time evolution of the maximum power point or with the photocurrent at maximum power point; see ref. 7 for details.*	[x] Yes [ ] No	Photocurrent tracked at maximum power point for about 400 min is provided in Supplementary Figure 3.

3. Hysteresis or any other unusual behaviour

Description of the unusual behaviour observed during the characterization	[x] Yes [ ] No	Supplementary Figure 6 shows there is no hysteresis during the characterization.
Related experimental data	[x] Yes [ ] No	Data shown in Supplementary Figure 6 in the supplementary file.

4. Efficiency

External quantum efficiency (EQE) or incident photons to current efficiency (IPCE)	[x] Yes [ ] No	EQE spectra of cells are shown in Supplementary Figure 2.
A comparison between the integrated response under the standard reference spectrum and the response measure under the simulator	[x] Yes [ ] No	Integrated EQE under AM1.5 is comparable to current density under solar simulator.
For tandem solar cells, the bias illumination and bias voltage used for each subcell	[ ] Yes [x] No	No tandem cells are reported in the work.

5. Calibration

Light source and reference cell or sensor used for the characterization	[x] Yes [ ] No	Described in "Methods" section. The light source is a solar simulator (Newport, Sol3A) .
Confirmation that the reference cell was calibrated and certified	[x] Yes [ ] No	Described in "Methods" section. The reference silicon cell was certified by NIST (Newport 532 ISO1599).

November 2017

Calculation of spectral mismatch between the reference cell and the devices under test

- [ ] Yes
- [x] No

Mismatch is not calculated.

6. Mask/aperture

Size of the mask/aperture used during testing

- [x] Yes
- [ ] No

Described in "Methods" section. Aperture area of 4.095 mm^2 was used.

Variation of the measured short-circuit current density with the mask/aperture area

- [x] Yes
- [ ] No

Described in "Methods" section. There is no variation.

7. Performance certification

Identity of the independent certification laboratory that confirmed the photovoltaic performance

- [ ] Yes
- [x] No

This work focuses on a mechanically robust new interlayer.

A copy of any certificate(s)
Provide in Supplementary Information

- [ ] Yes
- [x] No

N/A

8. Statistics

Number of solar cells tested

- [x] Yes
- [ ] No

12 to 28 cells for different types of cells were tested.

Statistical analysis of the device performance

- [x] Yes
- [ ] No

Statistical analysis are provided in Supplementary Figure 4.

9. Long-term stability analysis

Type of analysis, bias conditions and environmental conditions
For instance: illumination type, temperature, atmosphere humidity, encapsulation method, preconditioning temperature

- [x] Yes
- [ ] No

A cell is tested under one-sun solar simulation illumination for 200 h (Supplementary Figure 5).
